# A case of severe cerebral embolism after chemotherapy for HER2-positive gastric cancer

**DOI:** 10.1186/s13104-015-1059-2

**Published:** 2015-03-26

**Authors:** Takayuki Takahama, Masayuki Takeda, Shinichi Nishina, Kazuhiko Nakagawa

**Affiliations:** Department of Medical Oncology, Kinki University Faculty of Medicine, 377-2 Ohno-higashi, Osaka-Sayama, Osaka 589-8511 Japan

**Keywords:** Intracranial embolism, Chemotherapy, Gastric cancer, Human epidermal growth factor receptor 2

## Abstract

**Background:**

Trastuzumab + chemotherapy is considered the standard therapy for advanced human epidermal growth factor receptor 2 (HER2)-positive gastric cancer with mild manageable toxicity, on the basis of the results of a pivotal phase-III trial. Cerebrovascular events are not recognized as expected adverse effects of such therapy.

**Case presentation:**

We report the case of a 67-year-old, current-smoking male with stage-IV HER2-positive gastric cancer who suffered right middle cerebral artery (MCA) embolism after trastuzumab + chemotherapy. He received trastuzumab and cisplatin on Day 1, followed by a continuous 5-fluorouracil infusion for 5 days as a first-line treatment. Four days after chemotherapy initiation, he presented with left hemiplegia, and brain magnetic resonance imaging and magnetic resonance angiography revealed a right MCA occlusion. No further chemotherapy was administered because of worsening performance status.

**Conclusion:**

The present case, possibly the first such reported case, suggests the risk of development of embolism after trastuzumab + chemotherapy in HER2-positive advanced gastric cancer, although other factors should be considered.

## Background

Thromboembolism is a known vascular toxicity associated with tumor chemotherapy. The combination of trastuzumab and chemotherapy has shown significant antitumor activity, with mild manageable toxicity in patients with human epidermal growth factor receptor 2 (HER2)-positive advanced gastric cancer [1], whereas cerebral arterial embolism has not been recognized as a side effect expected with this regimen. We describe an unusual case of advanced gastric cancer, in which the patient developed right middle cerebral artery embolism after chemotherapy combined with trastuzumab.

## Case presentation

**Figure 1:**
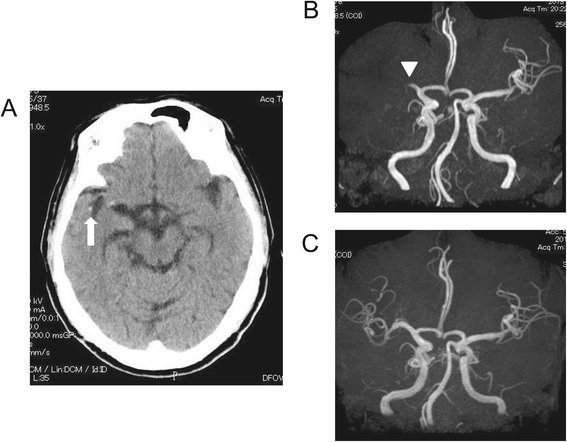
**Brain images after the onset of cerebral infarction. A**: Noncontrast brain computed tomography revealed a “dot” sign in the right middle cerebral artery (MCA; arrow) seen as a dot in the sylvian fissure. **B**: Brain magnetic resonance angiography (MRA) showing an occlusion of the right MCA (arrowhead) on the day of the stroke. **C**: Brain MRA 3 days after the stroke showing blood flow of the damaged area of the brain.

A 67-year-old man was diagnosed with stage-IV gastric cancer with multiple liver and lymph node metastases, but no apparent brain metastases, in April 2013. The patient had no previous medical history of arrhythmia, ischemic heart disease, diabetes mellitus, or stroke, and he was not taking any daily medications. An echocardiogram before chemotherapy demonstrated no cardiac thrombus, no atrial/ventricular septal defects, no patent foramen ovale, no valvular vegetations, and a left ventricular ejection fraction (LVEF) of 67%. Immunohistochemical analysis showed a HER2-positive score of 3+ for a primary tumor. Thus, the patient was treated with trastuzumab, and cisplatin on day 1, with subsequent continuous infusion of 5-fluorouracil (5-FU) for 5 days. Although the patient did not experience any adverse events at the start of chemotherapy, 4 days afterward, he noticed a sudden onset of left hemiplegia and agitation. Laboratory testing showed grade-1 anemia (hemoglobin, 12.5 g/dL), increased number of platelets (452,000/μL), healthy levels of fibrinogen (235 mg/dL; reference range 150–340 mg/dL) and the prothrombin time–international normalized ratio (PT-INR) of 1.05 (reference range 0.90–1.10), and a slight increase in fibrin degradation product (FDP) levels to 10.3 μg/mL; reference range 0–4.0 μg/mL). An electrocardiogram showed a healthy sinus rhythm with no ST–T changes. Noncontrast brain computed tomography (CT) revealed a “dot sign” in the right middle cerebral artery (MCA) seen as a dot in the sylvian fissure (Figure 1A), which is known as an early CT marker of acute cerebral infarction. Brain magnetic resonance imaging and magnetic resonance angiography revealed an MCA occlusion (Figure 1B), consistent with a diagnosis of chemotherapy-induced grade-4 stroke. After consultation with a neurologist, emergency endovascular therapy with an aspiration catheter and balloon was performed. Removal of the clot did restore the blood flow; however, distal emboli still remained (Figure 1C). The patient concomitantly received argatroban and aspirin. No further chemotherapy was administered because of the deterioration in the performance status of the patient. He underwent extensive neurorehabilitation, which brought a slight improvement in his neurological status. One month after initiation of chemotherapy, his carcinoembryonic antigen level decreased from 15.4 ng/mL to 7.6 ng/mL (reference range 0.0–5.0); however, he developed jaundice because of the tumor burden due to liver metastasis. His condition worsened and he died 2.0 months after chemotherapy initiation.

## Discussion and conclusions

The combination of trastuzumab and chemotherapy is considered the standard of care for patients with HER2-positive advanced gastric cancer on the basis of the results of a pivotal phase-III trial, which showed the efficacy of trastuzumab combined with cisplatin plus capecitabine or 5-FU as a first-line therapy [1]. The toxicity profile is mild, and no severe thromboembolisms such as cerebral infarction have been reported to date. In the present case, the patient presented with MCA embolism on the fourth day of the first cycle of chemotherapy with 5-FU, cisplatin, and trastuzumab. The mechanism underlying the cerebrovascular event caused by a chemotherapy regimen is likely multifactorial, including tumor microemboli and thromboembolism. The proposed mechanism of tumor embolization is invasion of the pulmonary veins, with or without left atrial invasion [2]. In rare cases, a tumor may invade the venous circulation and spread to the left side of the heart through a patent foramen ovale, leading to systemic tumor embolization. The risk of tumor embolization in the present case appeared to be low because a chest CT scan had revealed no evidence of intrathoracic metastasis from the gastric cancer, and patent foramen ovale was not detectable on the echocardiogram.

A possible explanation in the present case is that thromboembolism may be responsible for this cerebrovascular event, and several factors may be at work here. First, platelet activation, alteration of the clotting cascade, including hyperfibrinolysis, and disturbances of the prostacyclin–thromboxane homeostasis, increase the risk of thrombosis 4- to 6-fold in cancer patients compared with the general population [3]. Clinically significant disseminated intravascular coagulation is unlikely in the presence of healthy levels of fibrinogen and PT-INR; however, increased FDP levels may indicate the presence of a clot. Second, smoking likely contributes to an increased stroke risk via both acute effects on the risk of thrombus generation in narrowed arteries and via chronic effects related to an increased burden of atherosclerosis. Nevertheless, the present case had no other risk factors of stroke, such as hypertension, diabetes mellitus, hypercholesterolemia, and atrial fibrillation [4]. Third, the literature suggests that cisplatin-based chemotherapy has been associated with an increased risk of arterial thromboembolic events [5], although severe thromboembolism, similar to that in the present case, has not been reported as a side effect associated with this regimen. Fourth, congestive heart failure is associated with a relatively high risk of venous thromboembolism. Therefore, we should rule out the possibility of trastuzumab-related thromboembolism, which is associated with trastuzumab-related cardiotoxicity. Troponin I is a strong predictor of reduction in the LVEF in patients who received chemotherapy (mainly anthracycline-containing regimens). A study has demonstrated that troponin I is mainly detected in blood plasma soon after the first trastuzumab cycle among troponin-positive breast cancer patients who received trastuzumab-containing regimens [6-8]. These results suggest that trastuzumab-induced cardiotoxicity occurs in the early phase of chemotherapy. The present case had no signs or symptoms of congestive heart failure, and we did not perform further analysis such as echocardiography after the onset of cerebral infarction. Nonetheless, the possibility of trastuzumab-related thromboembolism due to cardiotoxicity should be taken into account in the present case.

Trastuzumab in combination with platinum-based chemotherapy is a treatment option in patients with HER2-positive advanced gastric or those with cancer of the gastroesophageal junction. The present case suggests the possible risk of the development of cerebral embolism after chemotherapy initiation, although other factors should be considered. Further research is needed to elucidate the mechanisms underlying the neurovascular adverse events.

## Patient consent

Written informed consent was obtained from the patient for publication of this case report and accompanying images before the patient received chemotherapy.
